# Hidden Behind a Veil: A Rare Case of Pulmonary Nocardiosis

**DOI:** 10.7759/cureus.38635

**Published:** 2023-05-06

**Authors:** Elizabeth George, Chandramouli M T, Nandakishore Baikunje, Nandu Nair

**Affiliations:** 1 Pulmonary Medicine, KS Hegde Medical Academy, Mangaluru, IND

**Keywords:** tuberculosis, infectious diseases, pulmonary medicine, nocardiosis, hiv aids

## Abstract

A 48-year-old male, a known case of seizure disorder, presented with complaints of cough for four months, which increased for two weeks, fever for two weeks and weight loss. Computed tomography (CT) scan of the thorax showed multiple heterogeneously enhancing lesions of bilateral lung fields predominantly in peribronchovascular distribution with enlarged, necrosed and conglomerated lymph nodes suggestive of infective etiology. On routine blood investigations, he was found to be reactive for the human immunodeficiency virus. He underwent bronchoscopy and bronchoalveolar lavage culture grew Nocardia. He was prescribed antibiotics based on susceptibility reports and the patient became symptomatically better after one month and was discharged.

## Introduction

Nocardiosis is a bacterial infection which predominantly affects the lungs, brain and skin. It is most common in adult males, especially in individuals with weakened immune system. It is caused by a bacterium of the genus *Nocardia*, most commonly* Nocardia asteroids* or *Nocardia brasiliensis*. Patients should receive antibiotic treatment for months to attain cure from this infection.

## Case presentation

**Figure 1:**
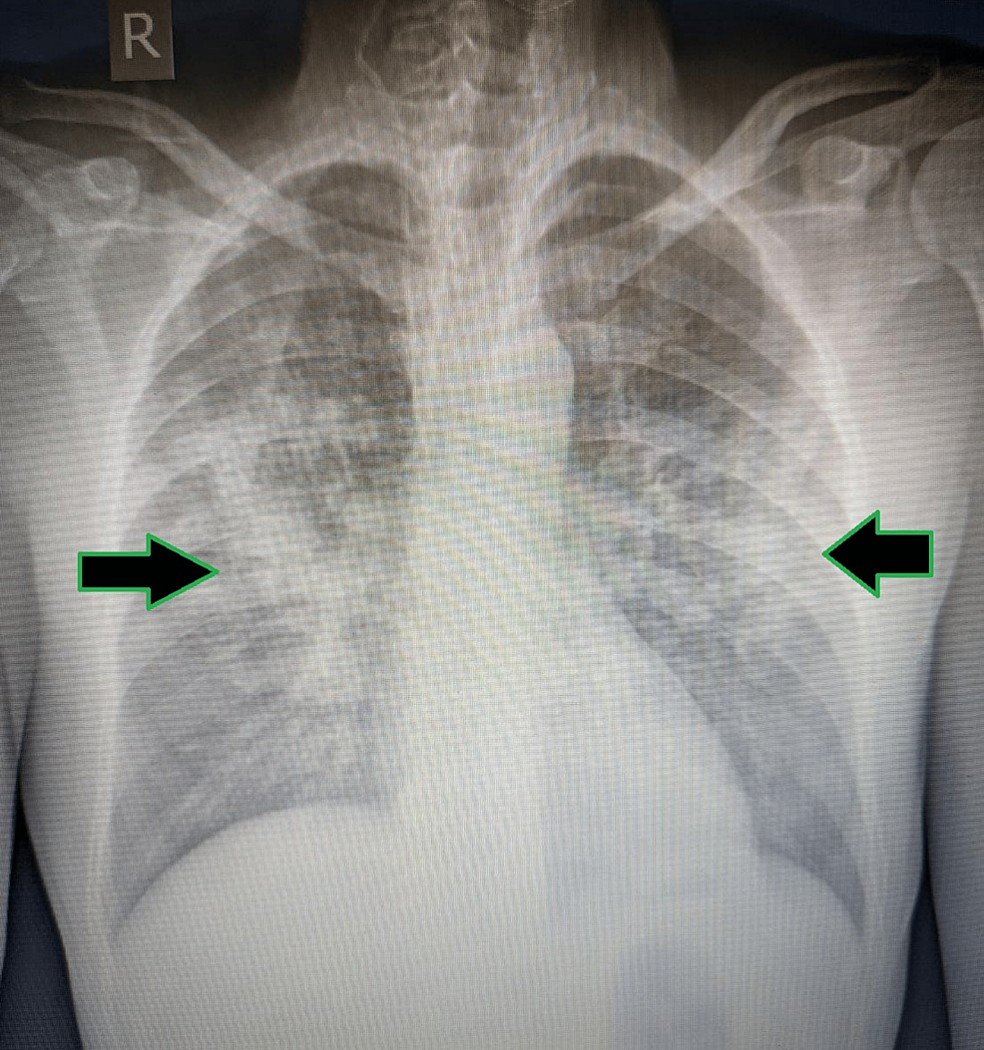
Initial chest X-ray showing bilateral bronchopneumonia (solid arrows with green outline).

A 48-year-old male with a history of seizure disorder presented to the Pulmonary Medicine outpatient unit with complaints of cough for four months which exacerbated for two weeks associated with scanty mucoid expectoration and fever for two weeks associated with chills and rigor with evening rise of temperature. The patient also had a history of weight loss of around 3 kg in one month. There was a history of non-projectile vomiting prior to hospital admission. The patient also had a history of chest pain following continuous coughing. There was no history of hemoptysis, foul-smelling sputum, breathlessness or orthopnea. A chest X-ray (CXR) was taken which showed bilateral infiltrates suggestive of bronchopneumonia (Figure 1).

**Figure 2:**
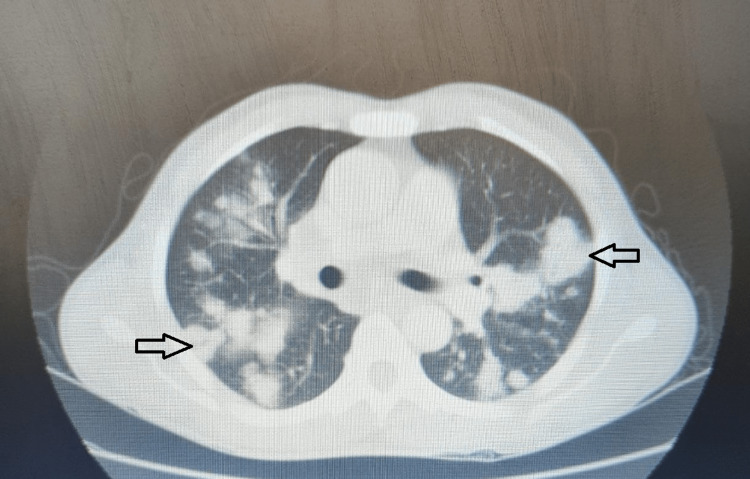
CT image showing ‘multiple heterogeneously enhancing lesions of bilateral lung fields’ in the lung window (arrows with black outline).

**Figure 3:**
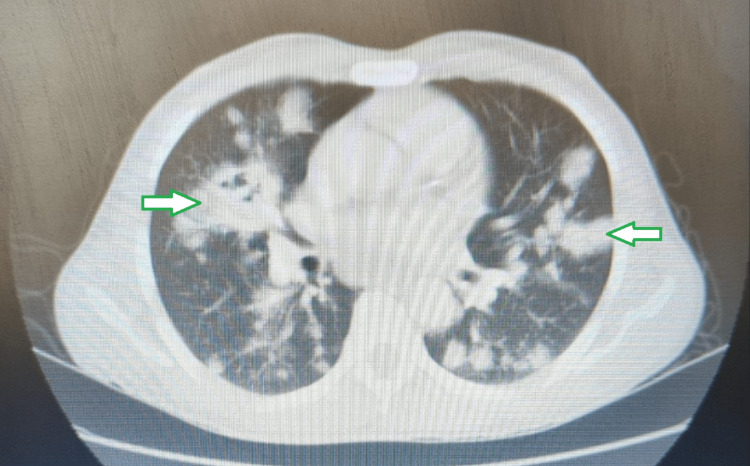
CT image showing ‘multiple heterogeneously enhancing lesions of bilateral lung fields’ in the lung window at a different area of the lung (solid arrows with green outline).

Sputum was sent for acid-fast bacilli (AFB) and culture and sensitivity which were both reported as negative. CT scan of the thorax was done and showed “multiple heterogeneously enhancing lesions of bilateral lung fields predominantly in peribronchovascular distribution with enlarged necrotic, conglomerated lymph nodes suggestive of infective etiology” (Figures 2, 3).

**Figure 4:**
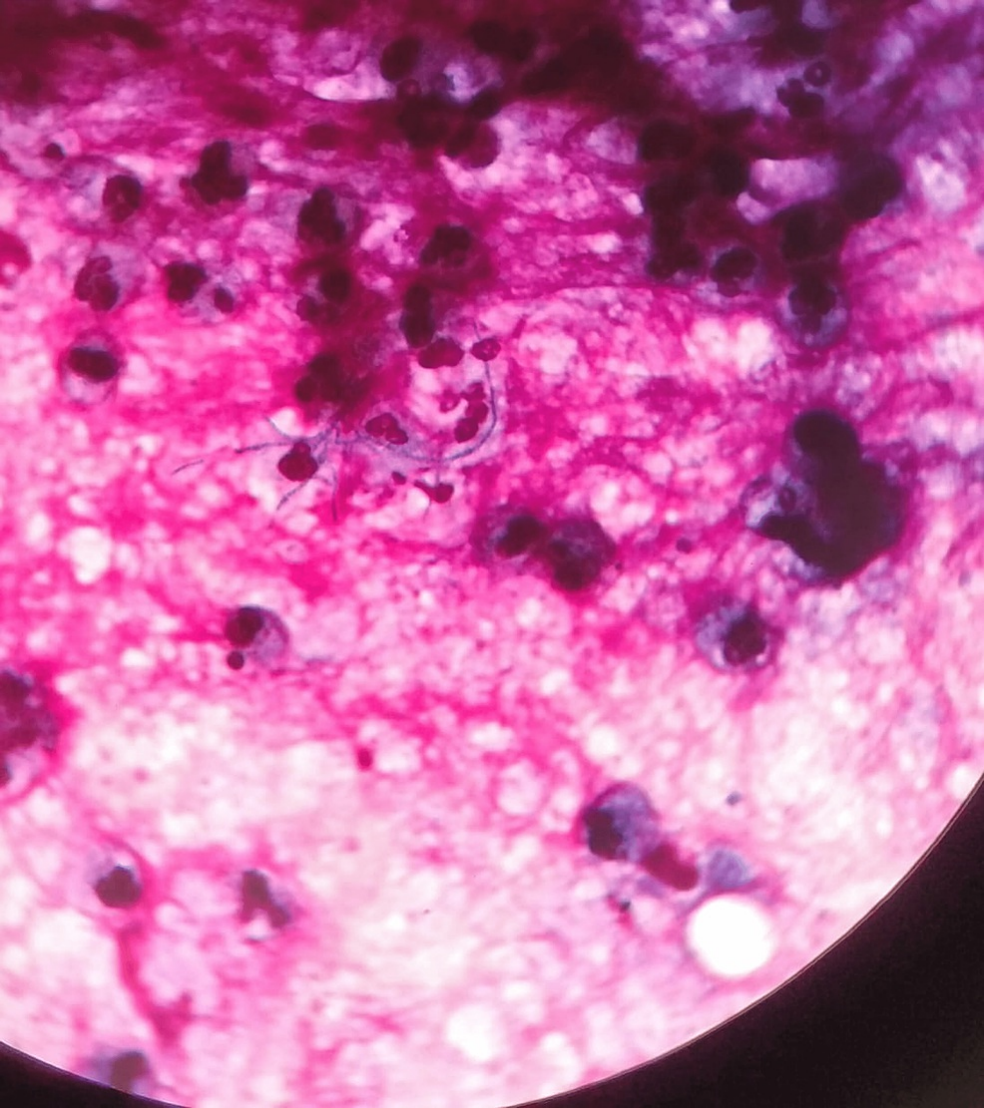
BAL gram staining in 100x magnification (oil immersion) showing gram-positive, filamentous bacilli.

The patient underwent bronchoscopy and bronchoalveolar lavage (BAL) was sent for further analysis. BAL gram staining showed filamentous, gram-positive bacilli (Figure 4).

**Figure 5:**
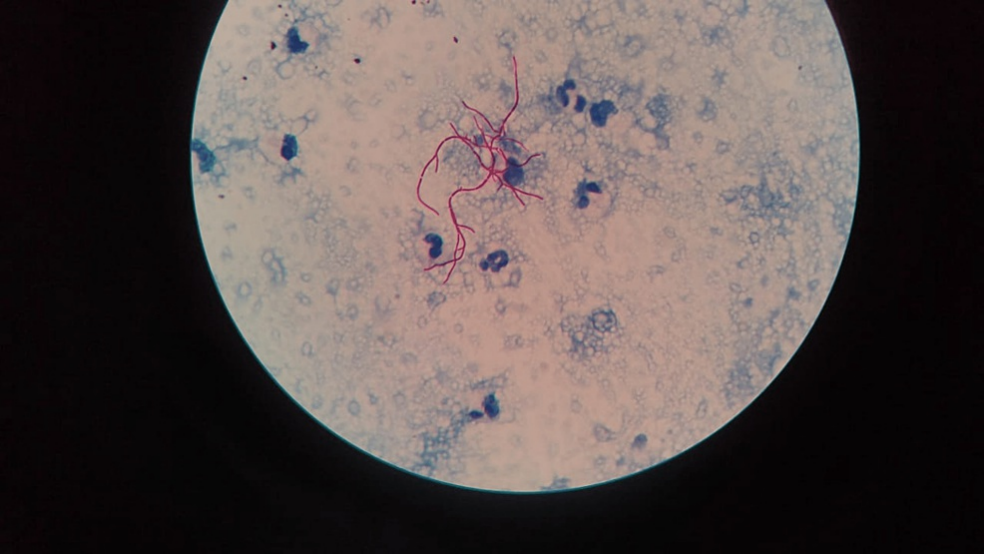
BAL-modified AFB staining in 100x magnification (oil immersion) showing filamentous, branching AFB resembling Nocardia. AFB: Acid-fast bacilli; BAL: Bronchoalveolar lavage

BAL-modified acid-fast testing showed filamentous, branching acid-fast bacilli (AFB) resembling Nocardia (Figure 5).

**Figure 6:**
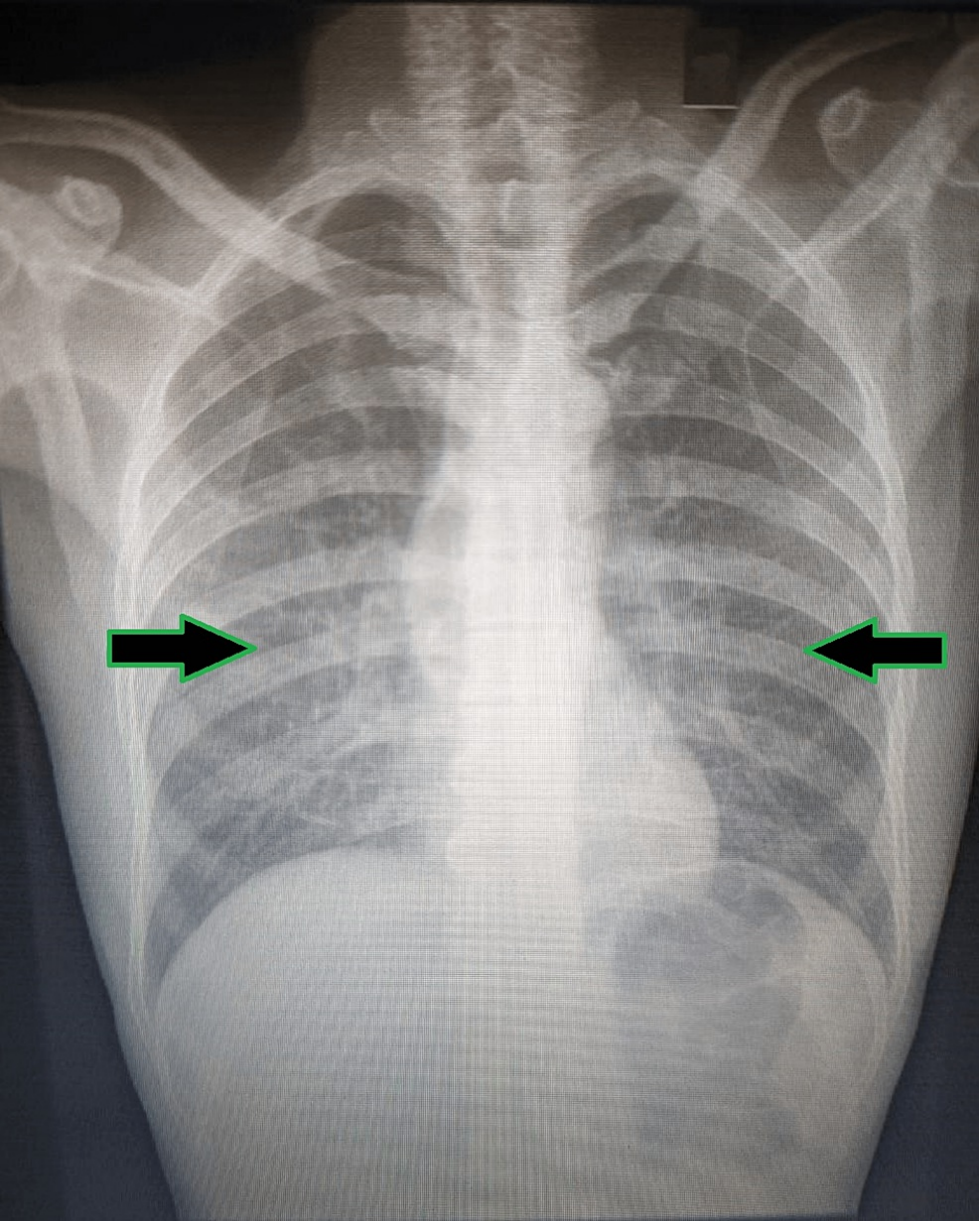
Chest X-ray taken at the time of discharge which shows the resolution of shadows when compared to the initial radiograph (solid arrows with green outline).

Mycobacterium tuberculosis (MTb) was not detected in the cartridge-based nucleic acid amplification test (CBNAAT) of BAL but grew Nocardia in bacterial culture. He was prescribed antibiotics. Blood investigations were done and were found to be reactive for the human immunodeficiency virus (HIV). The patient was started on anti-retroviral treatment and showed improvement in the form of reduction in symptoms as well as clearance of shadows on repeat CXR after one month (Figure 6).

## Discussion

Nocardiosis is an infectious disease caused by the gram-positive bacterium Nocardia spp. In contrast to other gram-positive bacteria, *Nocardia *appears on direct microscopy as a filamentous bacterium with hyphae-like branching. The modified Kinyoun acid-fast stain is employed to identify *Nocardia*. Nocardia appears under a microscope as “beaded” acid-fast. *Nocardia *can seem similar to *Actinomyces *species on Gram stains, however, *Actinomyces *species do not grow in anaerobic conditions and are not acid-fast.

The bulk of infections caused by *Nocardia*, an “opportunistic pathogen,” takes place in people with impaired cell-mediated immunity, such as people with acquired immunodeficiency syndrome (AIDS) or organ transplant recipients [1]. However, up to one-third of nocardiosis patients are immunocompetent [2]. Patients with lymphoma, certain other malignancies, HIV infection, solid organ or hematopoietic stem cell transplant, or those undergoing protracted treatment with steroids or other drugs that suppress cell-mediated immunity are particularly at risk for infection because they have impaired cell-mediated immunity [3,4].

The most frequent clinical manifestation of infection is pulmonary nocardiosis since inhalation is the main way that bacteria are exposed to humans. A productive or nonproductive cough, shortness of breath, chest pain, hemoptysis, fever, night sweats, weight loss, and progressive fatigue are just a few of the symptoms that may appear at any time, from the subacute stage to the more chronic ones. The chest radiograph can vary, showing cavitary lesions as well as nodular and/or consolidation infiltrates with localized or multifocal illness [5,6]. Approximately one-third of patients may develop pleural effusions. Clinically and radiographically, it can be very challenging to distinguish Nocardia from filamentous fungal (such as aspergillosis, mucormycosis) or mycobacterial disease. Nocardia may occasionally be found in the respiratory system of a person who does not appear to have a pulmonary infection. Patients with underlying lung diseases, such as bronchiectasis and cystic fibrosis, can experience *Nocardia *isolation without an obvious pulmonary infection [7], which is concerning and should be treated with caution. Never disregard the finding of *Nocardia *in any patient, especially if there are any unusual clinical or radiologic pulmonary abnormalities.

Extrapulmonary nocardiosis is a relatively frequent condition that can spread to the pericardium, pleura, mediastinum, and vena cava through hematogenous dissemination or adjacent necrotizing pneumonitis. Extrapulmonary nocardiosis is characterised by the formation of abscesses, which can first appear as a chronic granulomatous or mixed progressive inflammatory mass or as a pyogenic bacterial process. The most typical extrapulmonary site for nocardiosis is the central nervous system (CNS) (up to 44 percent in one series) [5]. Patients may exhibit headache, nausea, vomiting, convulsions, or alterations in consciousness in addition to having one or more brain abscesses [7]. Neurologic symptoms normally appear gradually, though occasionally they might present suddenly and advance quickly. Although isolated CNS disease can happen, cerebral nocardiosis frequently goes hand in hand with lung disease.

Our case report describes an HIV-positive immunocompromised individual who presented with symptoms similar to tuberculosis but was later proven to be infected with *Nocardia*.

This is a rare occurrence and shows to prove that not all acid-fast bacilli need to be taken as Mycobacteria, even in high-burden countries.

## Conclusions

This case report shows that bronchoscopy and BAL will help in diagnosing uncommon infective etiologies of the lung. Opportunistic infections have to be considered when working up a case with immunodeficiency. Nocardiosis can cause disseminated disease and multiple organs could be affected at the first presentation.

## References

[1] Martínez-Barricarte R (2020). Isolated nocardiosis, an unrecognized primary immunodeficiency?. Front Immunol.

[2] Beaman BL, Burnside J, Edwards B, Causey W (1976). Nocardial infections in the United States, 1972-1974. J Infect Dis.

[3] Young LS, Rubin RH (2002). Mycobacterial and nocardial diseases in the compromised host. In: Clinical Approach to Infection in the Compromised Host. 4th Ed..

[4] Long PF (1994). A retrospective study of Nocardia infections associated with the acquired immune deficiency syndrome (AIDS). Infection.

[5] Beaman BL, Beaman L (1994). Nocardia species: host-parasite relationships. Clin Microbiol Rev.

[6] Martínez R, Reyes S, Menéndez R (2008). Pulmonary nocardiosis: risk factors, clinical features, diagnosis and prognosis. Curr Opin Pulm Med.

[7] Brown-Elliott BA, Brown JM, Conville PS, Wallace RJ Jr (2006). Clinical and laboratory features of the Nocardia spp. based on current molecular taxonomy. Clin Microbiol Rev.

